# Supplementary material for: Inferring Drug-Protein–Side Effect Relationships from Biomedical Text
Source: Genes (Basel). 2019 Feb 19;10(2):159. doi: 10.3390/genes10020159 (PMC6409686; doi:10.3390/genes10020159)
Supplement: Supplementary file 1 [file genes-10-00159-s001.zip › supplementary/supplementary(Further_literature_analysis).docx]

**Further literature analysis**

***Type1 case - Path 7***

One example for ‘type1’ in which the ‘entities’ and ‘verbs’ are well connected is path 7. Path 7 shows the connection between the drug ‘Sorafenib’ and the side effect ‘dysepsia’.

Sorafneib is a kinase inhibitor drug which is used in the treatment for primary kidney cancer, and advanced primary liver cancer (1), (2). Sorafneib treatment can induce autophagy therefore inhibit tumor growth (3). Uncontrolled growth in many cancers is due to a defect in the Ras-Raf-MEK-ERK pathway also known as the MAP/ERK pathway (4). Sorafneib acts as an inhibitor for several tyrosine protein kinases. Tyrosine protein kinases such as such as VEGFR, PDGFR and Raf family kinases are inhibited by Sorafneib therefore resulting in the suppression of tumor growth (5). It has also been shown that Sorafenib can inhibit the activation of the MAP kinase p38 by a marked decrease in p38 phosphorylation without affecting total protein levels (6), (7). These findings supports the connection between ‘Sorafneib’ and ‘p38’ in which it is connected by the verbs ‘inhibit’ and ‘block’.

p38 mitogen-activated protein kinases is one of the main sub-groups of the MAP kinases which plays a vital role in signal transduction and numerous biological processes such as cell differentiation, apoptosis and senescence (8), (9), (10), (11). Activation of p38 has been shown to occur in response to extracellular stimuli such as UV light, heat, osmotic shock, inflammatory cytokines (TNF-alpha & IL-1), and growth factors (CSF-1) (8).

Gastrin-17 (G-17) also known as ‘little gastrin 1’ is a form of gastrin which is a protein hormone secreted by the intestine (12). Primarily three types of gastrin are found gastrin-34(big gastrin), gastrin-17(little gastrin), and gastrin-14(mini gastrin) (12). Gastrin is produced in the G cells of the duodenum and in the pyloric antrum of the stomach and is released in response to certain stimulus such as hypercalcemia (an elevated calcium level in the blood) (13), (14). Gastrin stimulates hydrochloric acid (HCl)/gastric acid secretion by inducing histamine release from the ECL cells, hence functions as a central regulator for gastric acid secretion (15). In our study we considered ‘gastrin-17’ as ‘gastrin’ combining the two entities as one due to gastrin-17 is a form of gastrin and all three form of gastrin are produced in the G cells as well the functions are similar. .

The connection between ‘p38’ and ‘gastrin-17/gastrin’ was connected by the verb ‘inhibit’. We could not find studies directly connecting from ‘p38’ to ‘gastrin’ while there were studies directly connecting from ‘gastrin’ to ‘p38’. We searched for other factors that can connect from ‘p38’ to ‘gastrin’ and found ‘NF-κB’. NF-κB is a protein complex that regulates transcription, cytokine production, cell survival and is involved in multiple cellular responses was found to be regulated by p38 in which the p38 MAP kinase activity regulated the transcriptional activation of NF- κB (16), (17) (18), (19), (20). p38 MAP kinase was found to up regulate NF-κBs transcriptional activation through RelA phosphorylation during stretch-induced myogenesis and also NF-κB activity was induced in C2C12 cells by the activation of p38 (20), (21). Gastrin was found to be regulated by NF-κB in which IL1B-activated NF-κB down-regulated gastrin and was found later that this down-regulation occurred both in the presence and absence of IL1B (22), (23). Ectopic expression of NF-κB p65(p65 a sub-unit of NF-κB) in AGS cells resulted in about nine-fold transcriptional repression of gastrin which shows that gastrin is negatively regulated by NF-κB (23). These findings suggest a connection from p38 to gastrin by NF-κB in which activation of p38 MAP kinase up regulates NF-κB and activated NF-κB represses the transcription of gastrin. Another factor except of NF-κB that can connect from ‘p38’ to ‘gastrin’ is ‘calcium’. Osteoclasts are a type of bone cells derived from the monocyte/macrophage haematopoietic lineage that breaks down bone tissue a process which is critical in bone maintenance, repair, and remodeling (24). It was found that p38 MAP kinase signaling plays a crucial role in PTHrP-induced osteoclastic bone resorption which is the process by which osteoclasts break down bone resulting in a transfer of calcium from bone fluid to the blood (25), (26). FR167653 which is an inhibitor for p38 MAP kinase was found to inhibit PTHrP-induced osteoclastogenesis in vitro and PTHrP-induced bone resorption in vivo (25). Also studies show bone resorption induced by IL-1- and TNF is mediated by p38 MAP kinase and p38 activity enhances osteoclast maturation and bone resorption in myeloma (27), (28). These findings suggest that p38 MAP kinase activity plays a crucial role in osteoclast maturation and bone resorption thereby can regulate calcium levels in blood. As mentioned above gastrin is released in response to hypercalcemia (an elevated calcium level in the blood) suggesting that ‘p38’ can regulate ‘gastrin’ through ‘calcium’ however, more study is needed to confirm the exact regulation in how ‘p38’ regulates ‘calcium’. Through NF-κB we can support the connection form ‘p38’ to ‘gastrin’ by the verb ‘inhibit’ in which p38 MAP kinase up regulates NF-κB resulting in the inhibition of gastrin. While through ‘calcium’ we can support the connection between ‘p38’ and ‘gastrin’ but cannot support the verb ‘inhibit’. Still through paper research we have found high correlation between ‘p38’ and ‘gastrin’ through ‘calcium’ showing that ‘calcium’ has a high chance to be a another mediator connecting ‘p38’ and ‘gastrin’.

Dyspepsia also known as indigestion, is a condition in which digestion is impaired. Dyspepsia is highly related with gastrin due to gastrin as mentioned above is a key regulator for gastric acid secretion a digestive fluid, formed in the stomach (15). Dyspepsia can be caused by Gastroesophageal reflux disease (GERD) a condition when stomach acid coming up from the stomach into the esophagus causes mucosal damage (29). One of the cause of mucosal damage can be harsh stomach acid (30). These findings supports the connection between ‘gastrin’ and ‘dyspepsia’ in which it is connected by the verb ‘associate’.

In short, we can suggest ‘dyspepsia’ as a side effect for ‘Sorafenib’ in which ‘Sorafenib’ inhibits ‘p38’ thereby inducing or regulating ‘gastrin’ which results in ‘dyspepsia’. Indeed one of the known side effect of ‘Sorafenib’ is ‘dyspepsia’ which supports our method of study (31). Through our method not only can we suggest ‘dyspepsia’ as a side effect for ‘Sorafenib’ but also can suggest a mechanism in which how ‘Sorafenib’ can cause ‘dyspepsia’ which was known through clinical trials.

***References***

1. Escudier, B; Eisen, T; Stadler, WM; Szczylik, C; Oudard, S; Siebels, M; Negrier, S; Chevreau, C; Solska, E; Desai, AA; Rolland, F; Demkow, T; Hutson, TE; Gore, M; Freeman, S; Schwartz, B; Shan, M; Simantov, R; Bukowski, RM (January 2007). "Sorafenib in advanced clear-cell renal-cell carcinoma". New England Journal of Medicine 356 (2): 125–34.
2. Keating GM, Santoro A (2009). "Sorafenib: a review of its use in advanced hepatocellular carcinoma". Drugs 69 (2): 223–40. doi:10.2165/00003495-200969020-00006. PMID 19228077.
3. Zhang Y (Jan 2014). "Screening of kinase inhibitors targeting BRAF for regulating autophagy based on kinase pathways.". J Mol Med Rep 9 (1): 83–90. doi:10.3892/mmr.2013.1781. PMID 24213221
4. Hilger RA, Scheulen ME, Strumberg D (December 2002). "The Ras-Raf-MEK-ERK pathway in the treatment of cancer" (PDF). Onkologie 25 (6): 511–8. doi:10.1159/000068621. PMID 12566895
5. Wilhelm SM, Adnane L, Newell P, Villanueva A, Llovet JM, Lynch M (October 2008). "Preclinical overview of sorafenib, a multikinase inhibitor that targets both Raf and VEGF and PDGF receptor tyrosine kinase signaling". Mol. Cancer Ther. 7 (10): 3129–40. doi:10.1158/1535-7163.MCT-08-0013. PMID 18852116
6. Rahmani, M., E. M. Davis, C. Bauer, P. Dent, and S. Grant. 2005. “Apoptosis induced by the kinase inhibitor BAY 43-9006 in human leukemia cells involves down-regulation of Mcl-1 through inhibition of translation”. J. Biol. Chem. 280:35217–35227
7. Edwards JP, Emens LA (Oct 2010). “The multikinase inhibitor sorafenib reverses the suppression of IL-12 and enhancement of IL-10 by PGE₂ in murine macrophages”. Int Immunopharmacol. (10):1220-8. doi: 10.1016/j.intimp.2010.07.002
8. Tyler ZARUBIN and Jiahuai HAN (2005), “Activation and signaling of the p38 MAP kinase pathway”. Cell Research 15, 11–18. doi:10.1038/sj.cr.7290257
9. Li Y, Jiang B, Ensign WY, Vogt PK, Han J. Myogenic differentiation requires signalling through both phosphatidylinositol 3-kinase and p38 MAP kinase. Cell Signal 2000; 12:751–7.
10. Xia Z, Dickens M, Raingeaud J, Davis RJ, Greenberg ME. Opposing effects of ERK and JNK-p38 MAP kinases on apoptosis. Science 1995; 270:1326–31.
11. Wang W, Chen JX, Liao R, et al. Sequential activation of the MEK-extracellular signal-regulated kinase and MKK3/6-p38 mitogen-activated protein kinase pathways mediates oncogenic ras-induced premature senescence. Mol Cell Biol 2002; 22:3389–403
12. Mutt, Viktor (2013-10-22). “Gastrointestinal Hormones: Advances in Metabolic Disorders”. Academic Press. ISBN 9781483215532
13. Abraham L. Kierszenbaum, Laura Tres (2016). “Histology and Cell Biology: An Introduction to Pathology”. ELSEVIER. ISBN 978-0-323-31330-8
14. Feng J, Petersen CD, Coy DH, Jiang JK, Thomas CJ, Pollak MR, Wank SA (2010). "Calcium-sensing receptor is a physiologic multimodal chemosensor regulating gastric G-cell growth and gastrin secretion". Proc. Natl. Acad. Sci. U.S.A. 107 (41): 17791–17796. doi:10.1073/pnas.1009078107. PMID 20876097
15. Waldum HL, Brenna E. Role of the enterochromaffin-like cells and histamine in the regulation of gastric acid secretion. Gastroenterol Clin Biol 1991; 15: 65C-72C.
16. Brasier AR (2006). "The NF-kappaB regulatory network". Cardiovascular Toxicology 6 (2): 111–30. doi:10.1385/CT:6:2:111. PMID 17303919
17. Gilmore TD (Oct 2006). "Introduction to NF-kappaB: players, pathways, perspectives". Oncogene 25 (51): 6680–4. doi:10.1038/sj.onc.1209954. PMID 17072321
18. Schulze-Osthoff K, Ferrari D, Riehemann K, Wesselborg S (Dec 1997). “Regulation of NF-kappa B activation by MAP kinase cascades”. Immunobiology.;198(1-3):35-49.
19. Olson CM, Hedrick MN, Izadi H, Bates TC, Olivera ER, Anguita J (Jan 2007). “p38 mitogen-activated protein kinase controls NF-kappaB transcriptional activation and tumor necrosis factor alpha production through RelA phosphorylation mediated by mitogen- and stress-activated protein kinase 1 in response to Borrelia burgdorferi antigens”. Infect Immun. (1):270-7. Epub 2006 Oct 30.
20. Ji G, Liu D, Liu J, Gao H, Yuan X, Shen G (Jan 2010). “p38 mitogen-activated protein kinase up-regulates NF-kappaB transcriptional activation through RelA phosphorylation during stretch-induced myogenesis”. Biochem Biophys Res Commun. 1;391(1):547-51. doi: 10.1016/j.bbrc.2009.11.095.
21. Bernat Baeza-Raja and Pura Muñoz-Cánoves (Apr, 2004). “p38 MAPK-induced Nuclear Factor-κB Activity Is Required for Skeletal Muscle Differentiation: Role of Interleukin-6”. Mol Biol Cell; 15(4): 2013–2026. doi: 10.1091/mbc.E03-08-0585
22. Chakravorty M, Datta De D, Choudhury A, Roychoudhury S. “IL1B promoter polymorphism regulates the expression of gastric acid stimulating hormone gastrin”. Int J Biochem Cell Biol. 2009;41:1502–1510
23. Datta De D, Datta A, Bhattacharjya S, Roychoudhury S (Aug 2013). “NF-kappaB mediated transcriptional repression of acid modifying hormone gastrin”. PLoS One;8(8):e73409. doi: 10.1371
24. William J. Boyle, W. Scott Simonet & David L. Lacey (May 2003). “Osteoclast differentiation and activation”. Nature 423, 337-342 | doi:10.1038/nature01658
25. Tao H, Okamoto M, Nishikawa M, Yoshikawa H, Myoui A (2011). “P38 mitogen-activated protein kinase inhibitor, FR167653, inhibits parathyroid hormone related protein-induced osteoclastogenesis and bone resorption”. PLoS One. 6(8):e23199. doi: 10.1371.
26. Teitelbaum SL. (2000). "Bone resorption by osteoclasts". Science 289 (5484): 1504–8. doi:10.1126/science.289.5484.1504. PMID 10968780
27. Kumar S, Votta BJ, Rieman DJ, Badger AM, Gowen M, Lee JC (Jun 2001). “IL-1- and TNF-induced bone resorption is mediated by p38 mitogen activated protein kinase”. J Cell Physiol;187(3):294-303.
28. He J, Liu Z, Zheng Y, Qian J, Li H, Lu Y, Xu J, Hong B, Zhang M, Lin P, Cai Z, Orlowski RZ, Kwak LW, Yi Q, Yang J (Dec 2012). “p38 MAPK in myeloma cells regulates osteoclast and osteoblast activity and induces bone destruction”. Cancer Res;72(24):6393-402. doi: 10.1158/0008-5472.
29. DeVault KR, Castell DO (1999). "Updated guidelines for the diagnosis and treatment of gastroesophageal reflux disease. The Practice Parameters Committee of the American College of Gastroenterology". Am J Gastroenterol 94 (6): 1434–42. doi:10.1111/j.1572-0241.1999.1123_a.x. PMID 10364004
30. Menges M, Müller M, Zeitz M (Feb 2001). “Increased acid and bile reflux in Barrett's esophagus compared to reflux esophagitis, and effect of proton pump inhibitor therapy”. Am J Gastroenterol. 96(2):331-7.
31. Marcia S. Brose, Catherine T. Frenette, Stephen M. Keefe, Stacey M. Stein (February 2014). “Management of Sorafenib-Related Adverse Events: A Clinician’s Perspective”. Seminars in Oncology. Volume 41, S1–S16

***Type2 case - Path 15***

One example for ‘type2’ in which the ‘entities’ are connected but the ‘verbs’ that describe the relation are not certain is path 15. Path 15 shows the connection between the drug ‘Nilotinib’ and the side effect ‘intracranial hemorrhage’. In our study ‘Nilotinib’ is first connected to ‘p38’ by the verb ‘increase’ and also ‘reduce’ which are opposite in meaning. Though we could not find how ‘Nilotinib’ connects to ‘p38’ by the verb ‘increase’ we found reason that the verb ‘reduce’ can connect between ‘Nilotinib’ and ‘p38’. Nilotinib is a small-molecule tyrosine kinase inhibitor which is used for the treatment of chronic myeloid leukaemia (a cancer of the white blood cells) (1). Nilotinib acts by selectively inhibiting Bcr-Abl kinase and thereby inhibits proliferation of Bcr-Abl expressing cells (2), (3). As described above p38 MAP kinase which plays a vital role in numerous biological process was shown to be inhibited by Nilotinib, in which Nilotinib blocked the activation of p38 MAP kinase by reducing the basal phosphorylation of p38 MAP kinase (4), (5). These findings suggest that the connection between ‘Nilotinib’ and ‘p38’ is by the verb ‘reduce’.

MAO-A also known as Monoamine oxidase A is a crucial regulator for normal brain function in which it degrades neuro-transmitters, such as dopamine, norepinephrine, and serotonin (6). In our study ‘p38’ is connected to ‘MAO-A’ by the verb ‘inhibit’. Indeed p38 MAP kinase inhibits MAO-A in which active p38 MAPK kinas results in the phosphorylation of the MAO-A protein and inhibition of MAO-A activity (7). Our study suggests that the connection from ‘MAO-A’ to ‘SSAO’ is by the verb ‘increase’. However we could not find the connection through the verb ‘increase’ rather the connection of ‘MAO-A’ and ‘SSAO’ was through interaction in which MAO-A interacts with VAP1 (another name for SAAO) (8). Though the relation through the verb ‘increase’ is not certain MAO-A and VAP1 are highly correlated in which VAP-1 and MAO-A are both flavin dependent amine oxidases therefore having similar function (9). Also through our literature review we could find both MAO-A and VAP1 can be regulated by ‘p38’ supporting MAO-A and VAP1 correlation. While ‘p38’ regulation of ‘MAO-A’ is as shown above the ‘p38’ regulation of ‘SSAO’ was through ‘NF-κB’. As described earlier ‘p38’ upregulates ‘NF-κB’. NF-κB controls VAP-1 gene expression and the nuclear translocation of NF kappa B is followed by induction of cell surface expression of VAP-1 (10) (11). These findings suggest ‘p38’ regulates ‘SSAO’ through ‘NF-κB’ in which p38’ upregulates ‘NF-κB’ resulting in the regulation of ‘SSAO’ by inducting SSAO cell surface expression and controlling SSAO gene expression.

Intracranial hemorrhage (ICH) is a bleeding within the skull. It can be caused when blood vessel within the skull is ruptured or leaks due to head injury, ruptured aneurysm, as well as disorders with blood clotting (12). It was shown that VAP-1/SSAO activity was significantly higher in patients who subsequently experienced parenchymal hemorrhage (a type of ICH) and elevated plasma VAP-1/SSAO activity was capable of predicting worse neurological outcome in these patients hence VAP-1/SSAO activity can function as predictor for intracranial hemorrhage (13). Furthermore, it was suggested that anti-VAP-1/SSAO drugs may prevent neurological worsening in patients with intracranial hemorrhage (13), (14). These findings support the connection between ‘SSAO’ and ‘Intracranial hemorrhage’ by the verb ‘predict’ in which VAP-1/SSAO activity can function as predictor for intracranial hemorrhage.

In short, ‘nilotinib’ reduces ‘p38’ thereby allow ‘MAO-A’ activity while the relation of ‘MAO-A’ and ‘SSAO’ are not well known reduction of ‘p38’ will also influence ‘SSAO’ regulation which can predict ‘intracranial hemorrhage’. Furthermore as NF-κB controls VAP-1/SSAO gene expression, reduction of p38 will lead to non-control of VAP-1/SSAO gene expression which can result in intracranial hemorrhage. Therefore through our method we can suggest ‘intracranial hemorrhage’ as a side effect for ‘nilotinib’. Like in the case of ‘Sorafenib’ and ‘dyspepsia’ one of the known side effect of ‘nilotinib’ was ‘intracranial hemorrhage’ therefore supports our method of study and suggest a mechanism in which how ‘nilotinib’ can cause ‘intracranial hemorrhage’ which was known through clinical trials (15), (16).

***References***

1. Gómez-Almaguer D, Saldaña-Vázquez R, Tarín-Arzaga L, Herrera-Rojas MA, de Larracoechea VM, Cantú-Rodríguez OG, Gutiérrez-Aguirre CH, Jaime-Pérez JC (Feb 2016). “Combination of low-dose imatinib plus nilotinib for the treatment of chronic-phase chronic myeloid leukaemia after imatinib failure”. Hematology, [Epub ahead of print]
2. Manley, P.; Stiefl, N.; Cowan-Jacob, S.; Kaufman, S.; Mestan, J.; Wartmann, M.; Wiesmann, M.; Woodman, R.; Gallagher, N. (2010). "Structural resemblances and comparisons of the relative pharmacological properties of imatinib and nilotinib". Bioorganic & Medicinal Chemistry 18 (19): 6977–6986. doi:10.1016/j.bmc.2010.08.026. PMID 20817538
3. Breccia, M.; Alimena, G. (2010). "Nilotinib: a second-generation tyrosine kinase inhibitor for chronic myeloid leukemia". Leukemia research 34 (2): 129–134
4. Villar VH, Vögler O, Martínez-Serra J, Ramos R, Calabuig-Fariñas S, Gutiérrez A, Barceló F, Martín-Broto J, Alemany R (2012). “Nilotinib counteracts P-glycoprotein-mediated multidrug resistance and synergizes the antitumoral effect of doxorubicin in soft tissue sarcomas”. PLoS One 7(5):e37735. doi: 10.1371
5. Ocuin LM, Zeng S, Cavnar MJ, Sorenson EC, Bamboat ZM, Greer JB, Kim TS, Popow R, DeMatteo RP (Oct 2012). “Nilotinib protects the murine liver from ischemia/reperfusion injury”. J Hepatol. 2012 (4):766-73. doi: 10.1016
6. Shih JC, Chen K, Ridd MJ (Jan-Feb 1999). “Role of MAO A and B in neurotransmitter metabolism and behavior”. Pol J Pharmacol 51(1):25-9.
7. Cao X, Rui L, Pennington PR, Chlan-Fourney J, Jiang Z, Wei Z, Li XM, Edmondson DE, Mousseau DD (Oct 2009). “Serine 209 resides within a putative p38(MAPK) consensus motif and regulates monoamine oxidase-A activity”. J Neurochem 111(1):101-10. doi: 10.1111
8. El-Maghrabey MH, Kishikawa N, Ohyama K, Imazato T, Ueki Y, Kuroda N (Jun 2015). "Determination of human serum semicarbazide-sensitive amine oxidase activity via flow injection analysis with fluorescence detection after online derivatization of the enzymatically produced benzaldehyde with 1,2-diaminoanthraquinone". Analytica Chimica Acta 881: 139–47. doi:10.1016/j.aca.2015.04.006. PMID 26041530
9. Januszewski AS, Mason N, Karschimkus CS, Rowley KG, Best JD, O'Neal DN, Jenkins AJ (May 2014). "Plasma semicarbazide-sensitive amine oxidase activity in type 1 diabetes is related to vascular and renal function but not to glycaemia". Diabetes & Vascular Disease Research 11 (4): 262–269. doi:10.1177/1479164114532963. PMID 24853908
10. Lockyer JM, Colladay JS, Alperin-Lea WL, Hammond T, Buda AJ (Feb 1998). “Inhibition of nuclear factor-kappaB-mediated adhesion molecule expression in human endothelial cells”. Circ Res. 23;82(3):314-20
11. Tozawa K, Sakurada S, Kohri K, Okamoto T (Sep 1995). “Effects of anti-nuclear factor kappa B reagents in blocking adhesion of human cancer cells to vascular endothelial cells”. Cancer Res 15;55(18):4162-7
12. Kushner D (1998). "Mild Traumatic Brain Injury: Toward Understanding Manifestations and Treatment". Archives of Internal Medicine 158 (15): 1617–1624. doi:10.1001/archinte.158.15.1617. PMID 9701095
13. Hernandez-Guillamon M, Garcia-Bonilla L, Solé M, Sosti V, Parés M, Campos M, Ortega-Aznar A, Domínguez C, Rubiera M, Ribó M, Quintana M, Molina CA, Alvarez-Sabín J, Rosell A, Unzeta M, Montaner J (Jul 2010). “Plasma VAP-1/SSAO activity predicts intracranial hemorrhages and adverse neurological outcome after tissue plasminogen activator treatment in stroke”. Stroke 41(7):1528-35. doi: 10.1161
14. Hernandez-Guillamon M, Solé M, Delgado P, García-Bonilla L, Giralt D, Boada C, Penalba A, García S, Flores A, Ribó M, Alvarez-Sabin J, Ortega-Aznar A, Unzeta M, Montaner J (2012). “VAP-1/SSAO plasma activity and brain expression in human hemorrhagic stroke”. Cerebrovasc Dis. 33(1):55-63. doi: 10.1159/000333370
15. RxList. Retrieved Nov. 15, 2015 from http://www.rxlist.com/tasigna-side-effects-drug-center.htm
16. Hazarika M, Jiang X, Liu Q, Lee SL, Ramchandani R, Garnett C, Orr MS, Sridhara R, Booth B, Leighton JK, Timmer W, Harapanhalli R, Dagher R, Justice R, Pazdur R (Sep 2008). “Tasigna for chronic and accelerated phase Philadelphia chromosome--positive chronic myelogenous leukemia resistant to or intolerant of imatinib”. Clin Cancer Res 1;14(17):5325-31. doi: 10.1158/1078-0432.

.

***Type3 case - Path 2***

One example for ‘type3’ in which the connection of ‘entities’ are difficult to interpret and also the ‘verbs’ that describe the relation are not certain is path 13. In path 13 the drug ‘anastrozole’ is connected to the side effect ‘acute hepatitis’. The first connection was between ‘anastrozole’ and ‘age’ by the verb ‘differ’. When considering the term ‘age’ as advanced glycation end-product (age) we could not find connections to ‘anastrozole’ by the verb ‘differ’ nor could we find any relations between ‘anastrozole’ and ‘advanced glycation end-product (age)’. Due to the verbs between ‘age’ which are ‘differ’ and ‘associate’ we considered ‘age’ as the term ‘life time’. By considering ‘age’ as ‘life time’ we could associate ‘anastrozole’ with ‘age’ but due to the broad term of ‘life time’ it was difficult to interpret the connection between ‘anastrozole’ and ‘age’ and also between ‘age’ and ‘MUC5AC’. We then considered if ‘anastrozole’ connects to ‘MUC5AC’ the third entity in our suggested path. Though we could not find direct connection or relation between ‘anastrozole’ and ‘MUC5AC’ we could connect both entities through ‘Androgen receptor (ar)’ which is also suggested in our study in path 14. ‘Anastrozole’ functions by binding reversibly to the aromatase enzyme, resulting in inhibition of the conversion from androgens to estrogens therefore supports and suggests connection form ‘Anastrozole’ to ‘Androgen receptor (ar)’ (1), (2). ‘Androgen receptor (ar)’ is not directly connected or related to ‘MUC5AC’ but can be through NF-kB. AR activation has been shown to cause a decrease in nuclear localization and transcriptional activity while increased NF-kB activity is shown to be associated with increased transcriptional activity of the MUC5AC promoter (3), (4). These findings can suggest that the connection between ‘anastrozole’ and ‘MUC5AC’ is possible through ‘androgen receptor (ar)’ and that the connection between ‘androgen receptor (ar)’ and ‘MUC5AC’ can be through NF-kB. Though it is possible to connect ‘anastrozole’ and ‘MUC5AC’ through our suggestion this can be done when ‘age’ is discarded from the suggested path. ‘Age’ as described above is difficult to connect with ‘anastrozole’ our first entity and ‘MUC5AC’ our third entity therefore is separated from the connections forming a gap in our suggested path.

***References***

1. Simpson ER (2003). "Sources of estrogen and their importance". The Journal of Steroid Biochemistry and Molecular Biology 86 (3–5): 225–30. doi:10.1016/S0960-0760(03)00360-1. PMID 14623515
2. Simpson ER (September 2003). "Sources of estrogen and their importance". J. Steroid Biochem. Mol. Biol. 86 (3-5): 225–30. doi:10.1016/S0960-0760(03)00360-1. PMID 14623515
3. Nelius T, Filleur S, Yemelyanov A, Budunova I, Shroff E, Mirochnik Y, Aurora A, Veliceasa D, Xiao W, Wang Z, Volpert OV (Sep 2007). “Androgen receptor targets NFkappaB and TSP1 to suppress prostate tumor growth in vivo’. Int J Cancer 1;121(5):999-1008.
4. Song JS, Cho KS, Yoon HK, Moon HS, Park SH (Dec 2005). “Neutrophil elastase causes MUC5AC mucin synthesis via EGF receptor, ERK and NF-kB pathways in A549 cells”. Korean J Intern Med. 20(4):275-83
