# Supplementary material for: Inferring Drug-Protein–Side Effect Relationships from Biomedical Text
Source: Genes (Basel). 2019 Feb 19;10(2):159. doi: 10.3390/genes10020159 (PMC6409686; doi:10.3390/genes10020159)

Path1(Type2) and Path2(Type3)

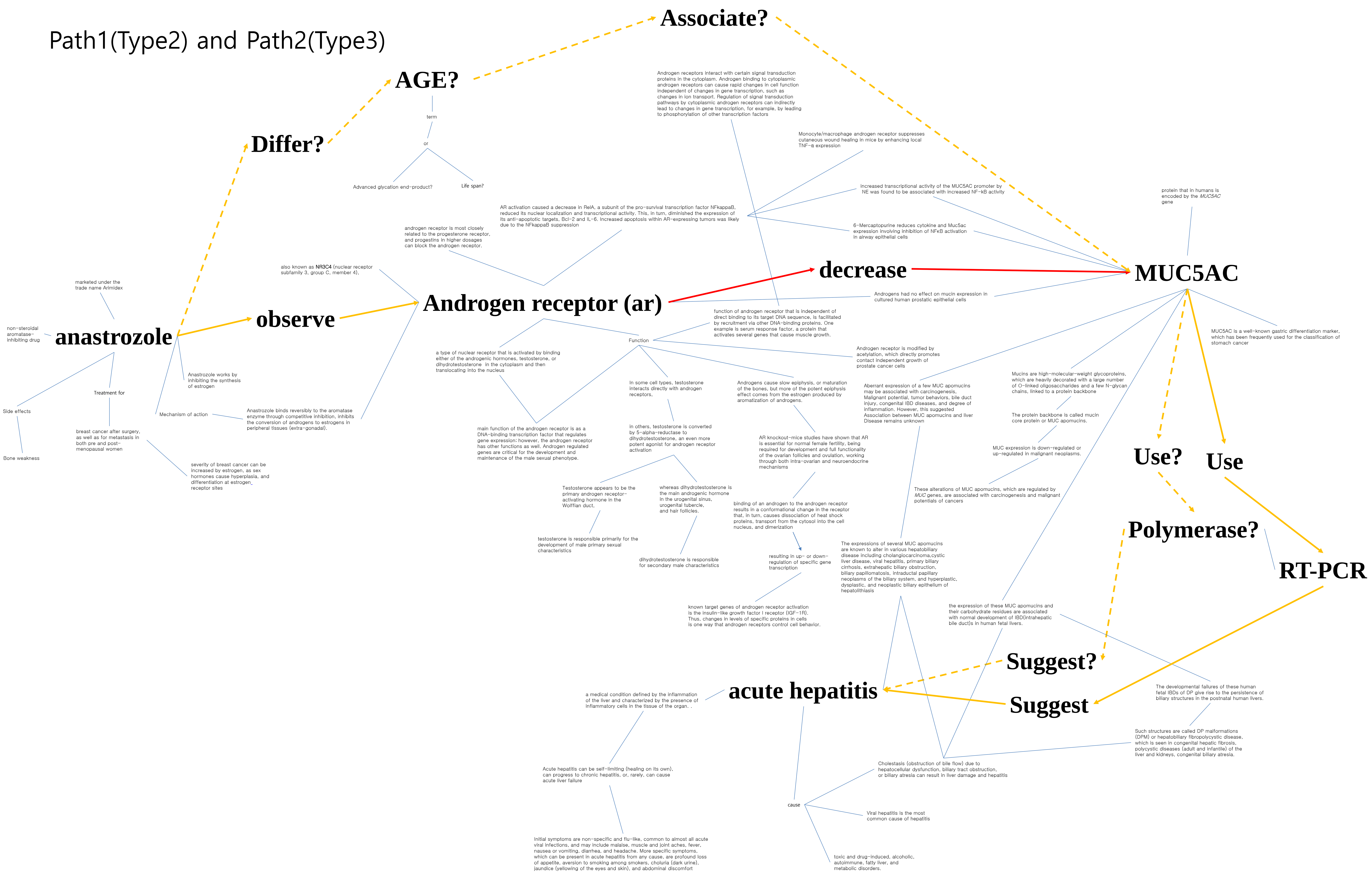

path 15(Type2)

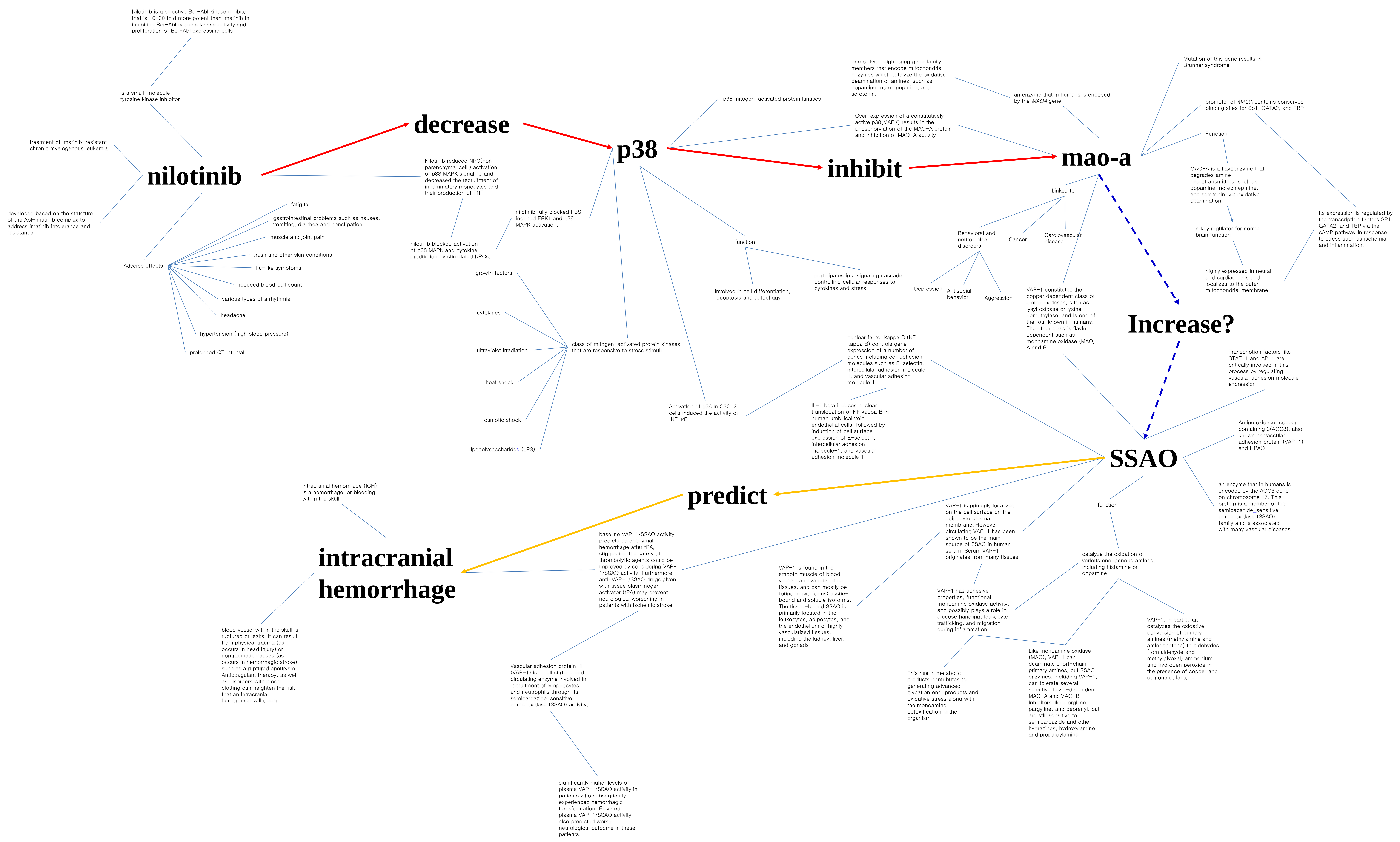

Supplement: Supplementary file 1 [file genes-10-00159-s001.zip › supplementary/supplementrary(image-path1&2&15).pdf]
